# Discharge guidance and telephone follow-up in the therapeutic adherence of heart failure: randomized clinical trial*

**DOI:** 10.1590/1518-8345.2484.3159

**Published:** 2019-08-19

**Authors:** Monica Isabelle Lopes Oscalices, Meiry Fernanda Pinto Okuno, Maria Carolina Barbosa Teixeira Lopes, Cassia Regina Vancini Campanharo, Ruth Ester Assayag Batista

**Affiliations:** 1 Universidade Federal de São Paulo, Escola Paulista de Enfermagem, São Paulo, SP, Brasil.; 2 Instituto Dante Pazzanese de Cardiologia, Pronto Socorro, São Paulo, SP, Brasil.

**Keywords:** Heart Failure, Education, Clinical Trial, Nursing, Emergency Nursing, Patient’s Discharge, Insuficiência Cardíaca, Educação, Ensaio Clínico, Enfermagem, Enfermagem em Emergência, Alta do Paciente, Insuficiencia Cardíaca, Educación, Ensayo Clínico, Enfermería, Enfermería de Urgencia, Alta del Paciente

## Abstract

**Objective:**

to evaluate the effectiveness of the behavioral intervention of discharge guidance and telephone follow-up in the therapeutic adherence, re-hospitalization and mortality of patients with heart failure.

**Method:**

randomized clinical trial without blinding, including 201 patients diagnosed with heart failure admitted to the emergency room, who were randomized in Control Group and Intervention Group. Intervention was carried out with specific discharge guidance in the Intervention Group, who were contacted for solving doubts via phone calls after 7 and 30 days, and the adherence to treatment was evaluated after 90 days with the Morisky test, the Brief Medical Questionnaire and the non-drug adherence test in both groups. The Generalized Estimating Equations Model was used (p<0.05%).

**Results:**

One-hundred and one patients were randomly sorted in the Control Group and in the Intervention Group, their average age being 62.6±15.2. The Intervention Group had higher drug and non-drug therapeutic adherence compared to the Control Group (p<0.001) and there were lower re-hospitalization and death rates in the Intervention Group after 90 days.

**Conclusion:**

discharge guidance with telephone follow-up was effective and resulted in greater therapeutic adherence, as well as in decrease of re-hospitalization and death rates in patients with heart failure. Clinical Trial Registration (REBEC): RBR- 37n859

## Abstract (pt)

**Objetivo:**

avaliar a efetividade da intervenção comportamental de orientação de alta e contato telefônico na adesão terapêutica, re-hospitalizações e mortalidade em pacientes com insuficiência cardíaca.

**Método:**

ensaio clínico randomizado, sem cegamento, incluídos 201 pacientes, internados em pronto socorro, com diagnóstico de insuficiência cardíaca. Randomizados em Grupo Controle e Grupo Intervenção, realizada intervenção de orientação de alta específica no Grupo Intervenção, contatados para reorientação via telefonemas em 07 e 30 dias e após 90 dias avaliado a adesão ao tratamento através dos testes de Morisky, *Brief Medical Questionnaire* e teste para adesão não medicamentosa em ambos grupos. Utilizou-se o Modelo de Equações de Estimação Generalizadas (p<0,05%).

**Resultados:**

foram alocados aleatoriamente 101 pacientes no Grupo Controle e 100 no Grupo Intervenção, com média de idade de 62,6±15,2. O Grupo Intervenção apresentou maior adesão terapêutica medicamentosa e não medicamentosa em relação ao Grupo Controle (p<0,001) e houve menor índice de re-hospitalização e morte no Grupo Intervenção após 90 dias.

**Conclusão:**

a orientação de alta com acompanhamento telefônico foi efetiva e resultou em maior adesão terapêutica, diminuição de re-hospitalizações e óbitos em pacientes com insuficiência cardíaca. Registro de Ensaio Clínico (REBEC): RBR- 37n859

## Abstract (es)

**Objetivo:**

evaluar la efectividad de la intervención conductual de orientación del alta y seguimiento telefónico en la adhesión terapéutica, las rehospitalizaciones y la mortalidad en pacientes con insuficiencia cardíaca.

**Método:**

ensayo clínico aleatorizado, no ciego, en el cual participaron 201 pacientes internados en la sala de urgencias, con el diagnóstico de insuficiencia cardíaca. Divididos al azar en Grupo Control y Grupo Intervención, se realizó una intervención de orientación de alta específica en el Grupo Intervención, siendo contactado por teléfono para la reorientación en 07 y 30 días después del alta; y después de 90 días se evaluó la adhesión al tratamiento por medio de las pruebas de Morisky, *Brief Medical Questionnaire* y la prueba para la adhesión no medicamentosa en ambos grupos. Se utilizó el Modelo de Ecuaciones de Estimación Generalizadas (p<0,05%).

**Resultados:**

fueron asignados aleatoriamente 101 pacientes en el Grupo Control y 100 en el Grupo Intervención, con un promedio de edad de 62,6±15,2. El Grupo Intervención tuvo una mayor adhesión terapéutica medicamentosa y no medicamentosa en comparación al Grupo Control (p<0,001), y hubo menores tasas de rehospitalizaciones y de fallecimientos en el Grupo Intervención después de 90 días del alta.

**Conclusión:**

la orientación del alta y el seguimiento telefónico se mostraron eficaces, promoviendo una mayor adhesión terapéutica, una disminución de las rehospitalizaciones y de fallecimientos en pacientes con insuficiencia cardíaca. Registro de Ensayo Clínico (REBEC): RBR- 37n859

## Introduction

Heart Failure (HF) represents a problem with great magnitude, as it is the second most common cause of death due to cardiovascular diseases in Brazil. The demand for emergency services on the part of this population is frequent, and the rates of hospitalization due to clinical complications are high, this having been a recurring public health problem for almost 20 years^1-3^.

The high number of hospital admissions due to HF, nearly one million annual hospitalizations, is commonly associated with decompensation of the disease, which can be triggered by cardiovascular factors, like ischemia and arrhythmias, and non-cardiovascular factors, like various types of infection. Another important factor in the clinical decompensation of these patients is the non-adherence to the treatment recommended for the disease^4-5^.

It is estimated that 50% of patients with chronic diseases do not adhere to treatment, due to voluntary of involuntary factors such as not knowing the medications, not understanding the medical prescription, lack of belief in the treatment and the patients’ psychosocial conditions^6^.

Discharge guidance is an important factor in the improvement of the patient’s understanding of the disease and of adherence to treatment. For an effective discharge guidance, it is important that it is conducted individually and according to the patients’ understanding of their disease^7-8^.

Studies reinforce the association of education in health with the improvement of the patients’ knowledge about HF, and the nurse plays an important role in the patient’s education and adherence to treatment^9-10^.

The literature demonstrates that telephone follow-up has been efficient, because patients who received calls after discharge that were intended to solve doubts, control signs and symptoms and guide treatment, adhered more to the therapy proposed, and there was decrease of the demand for care in the emergency units, as well as of the re-hospitalization and death rates^11-12^.

The high rate of re-hospitalization due to decompensation of HF leads to large expenses for the health system, in addition to increasing these patients’ morbidity and mortality^3^. In this context, the use of strategies that can substantially increase these individuals’ adherence to treatment should be considered by the inter-professional health team. Individualized hospital discharge guidance and telephone follow-up appear as strategies in health that can be performed by the nurse to optimize self-care^11-12^, improving the therapeutic adherence of patients with HF and decreasing the demand for hospital care.

Given the above, the purpose of this study was to verify whether a behavioral strategy consisting of telephone follow-up and individualized guidance given by the nurse contributes to increasing the drug adherence of patients with HF in a scenario where national research addressing this subject is still scarce. In this context, this study aimed to evaluate the effectiveness of the behavioral intervention of discharge guidance and telephone follow-up in the therapeutic adherence, re-hospitalization and mortality rates of patients with heart failure.

## Method

This was a randomized clinical trial, with 201 participants, drawn electronically and without blinding.

The study was conducted in 2016, between January and December of this year, in the emergency room of a public institution specialized in cardiology, with six emergency beds, 21 observation beds and 40 backup beds.

Patients admitted to the emergency room with a diagnosis of decompensated heart failure, registered on the medical records at the time of admission, with other associated diagnoses or not, over 18 years of age, who had not been subjected to myocardial revascularization surgery in the past 30 days and who could be contacted by telephone were included.

Sample size was determined with 80% power, 15% clinical difference and 5% clinical significance level, with statistical analysis of the questionnaires used. In this way, the total sample consisted of 201 patients.

The intervention encompassed specific and individualized discharge guidance for patients with HF on the verge of hospital discharge.

Numbers between one and 201 were electronically drawn with the aid of an online software, being randomly sorted in the Control Group (CG) and in the Intervention Group (IG). The patients were numbered sequentially at the time of hospital discharge in ascending order, from one to 201, and then allocated to either the CG or the IG according to their number, so there was no need for the use of sealed envelopes. The IG was composed of 100 participants and the CG, of 101.

To reduce the risk of bias, all interventions were carried out by a single researcher.

The sector’s nursing staff gave the CG an explanatory brochure standardized by the institution with the common discharge guidelines, containing information about dietary restrictions, recommended exercises, importance of drug adherence and return to the emergency service in case of worsening of the signs and symptoms. Three months after hospital discharge, the CG was contacted via telephone by the researcher, who evaluated their adherence to and the barriers to non-adherence to pharmacological measures, their adherence to non-pharmacological measures, as well as the occurrence of re-hospitalization and death in the period. The call was, on average, 15 minutes long.

The IG was given discharge guidance by the researcher, on the bed of the hospitalized patients and in the presence of whoever was accompanying them at that moment. As a first step, the researcher evaluated the patient’s previous knowledge regarding the disease and treatment, with a structured questionnaire based on the literature^1,8,13^.

Guidance started with the presentation to the patient of an illustrative video, of the Brazilian Association of Heart Failure, on the definition of HF. Then, individualized discharge guidance was performed according to the needs of each patient, with the reading of explanatory brochures. The brochures were devised according to the chronic heart failure guidelines of the Brazilian Society of Cardiology (SBC)^1,9,13^, and contained information about the definition of the disease, nutritional aspects, water restrictions and drug treatment.

The guidance in the IG followed a list of items to be addressed and was performed while taking into consideration the patients’ prior knowledge and their questions. The list was composed of the following items: i) the patient’s knowledge about the disease and treatment; ii) need of maintenance of water, caloric and sodium restriction; iii) performance of physical activity, if prescribed; iv) need to control weight; v) knowledge about the signs and symptoms of HF decompensation; and vi) importance of outpatient follow-up.

During guidance, the researcher had the medical prescription in hand to solve the doubts of the patients and their families regarding the use of drugs and possible side effects. The interviews lasted 20 minutes on average.

Thirty days after hospital discharge, the IG patients were contacted via telephone by the researcher to clarify doubts and to identify difficulties regarding treatment. In addition, it was identified if there had been cases of re-hospitalization or death during this period. The calls were, on average, 10 minutes long.

The evaluation of the effectiveness of the intervention in the IG was performed 90 days after hospital discharge, by telephone.

Therapeutic adherence based on drug and non-drug adherence and on the barriers for non-adherence were considered as primary outcomes, and the secondary outcomes included the occurrence of re-hospitalization and death seven, 30 and 90 days after hospital discharge.

With the aim of characterizing the study’s patients, an instrument with sociodemographic and clinical variables was devised, including: age, sex, education, length of hospitalization, marital status, occupation, weight, height, waist circumference, past occurrences and personal habits, and physical activity.

The consumption of alcohol was considered excessive when it exceeded 30 g alcohol/day, the equivalent of 625 ml of beer (~6% alcohol), 312.5 ml of wine (~12% alcohol) or 93.7 ml of distillates (~40% alcohol)^14^.

Physical activity was classified as follows: physically active individuals were considered to be those who practiced aerobic physical activities such as walking, running, cycling, dancing or swimming, three to five times a week for 30 to 60 minutes^13^.

The Morisky Green Test (MGT) was used to assess the patient’s adherence to the drug treatment, and consists of four questions: 1) Do you sometimes have trouble remembering to take your medication? 2) Do you sometimes neglect taking your medication? 3) When you are feeling better, do you sometimes stop taking your medication? 4) Sometimes, if you feel worse while taking the medication, do you stop taking it? The patient was classified as having “high adherence” when the answers to the four questions were negative; when one or two answers were positive, the patient was classified as having “moderate adherence”, and with three or four positive answers, he/she was classified as having “low adherence”^14^.

To identify the barriers to adherence to treatment, from the patient’s perspective, the Brief Medication Questionnaire (BMQ) was used. This instrument is divided into three domains: The Regimen Domain, which evaluates the patient’s behavior in relation to his/her adherence to the prescribed treatment; the Belief Domain, which assesses the patient’s belief in the treatment’s effectiveness and opinions about the unwanted side effects; and the Memory Domain, which identifies problems associated with remembering to take the medication. The presence of positive answers in any of the domains identifies a barrier to the treatment regimen prescribed^14^.

To evaluate adherence to non-drug treatment, a questionnaire was devised including three questions: 1) Do you control your weight as instructed by the health care professional? 2) Do you control your salt intake as instructed by the health care professional? 3) Do you control your water intake as instructed by the health care professional? The patient was classified as having “high adherence” when the answers to the three questions were positive; when one or two answers were positive, the patient was classified as having “moderate adherence”, and with three negative answers, he/she was classified as having “low adherence”^1^.

**Figure 1:**
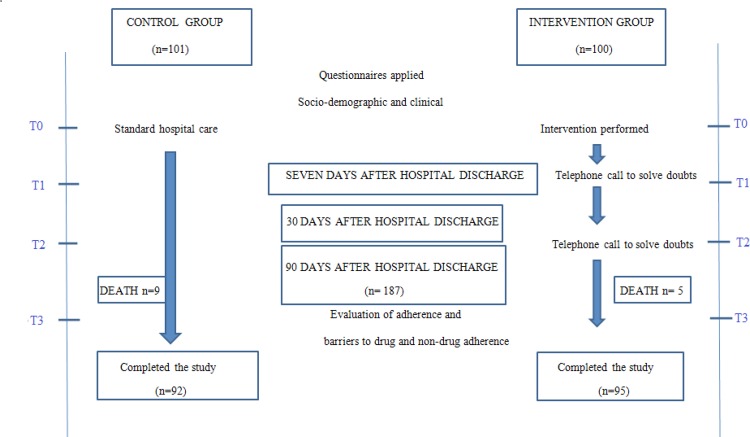
– Data collection flowchart. São Paulo, SP, Brazil, 2016

Data collection was divided into four phases: 1) at the time of hospital discharge in both groups (Time 0 – T0); 2) after seven days of hospital discharge, by telephone in the IG, and based on the records in the CG (Time 1 – T1); 3) after 30 days of hospital discharge, by telephone in the IG, and based on the records in the CG (Time 2 – T2); and 4) after 90 days of hospital discharge, by telephone in both groups (Time 3 – T3). The study’s data collection flowchart is presented in Figure 1.

The data were stored in spreadsheets in Excel, version 2007, and were analyzed by the SPSS statistical software version 19.

The independent variable was considered to be the behavioral intervention with discharge guidance and telephone follow-up.

The variables considered as dependent were: drug adherence, the barriers to non-adherence, non drug adherence, the occurrence of re-hospitalization and death. The control variables were: sex, age and comorbidities.

For the continuous variables, mean, standard deviation, median, minimum and maximum were estimated. For the categorical variables, frequency, percentage, and relative risk were estimated.

To compare drug adherence, the barriers to non-adherence and non-adherence 90 days after discharge, re-hospitalization and death seven, 30 and 90 days after discharge, between the groups, the following tests were used: Chi-Square Test, when necessary, Fisher’s Exact Test and the Generalized Estimating Equations Model, which aims to estimate regression parameters with correlated data, evaluate the relationship between the response and prediction variables in a population context, as well as the difference in the population’s mean response between two groups with different risk factors.

The homogeneity of the groups in relation to the socio-demographic and clinical variables was estimated using the Chi-Square Test or the Likelihood-Ratio Test and, for the continuous variables, the Mann-Whitney Test was used.

The significance level considered in all analyses was 5% (p-value < 0.05%)

The study was approved by the Research Ethics Committee of the Federal University of São Paulo (Unifesp), and registered in the Brazilian Registry of Clinical Trials: RBR-37n859.

**Figure 2:**
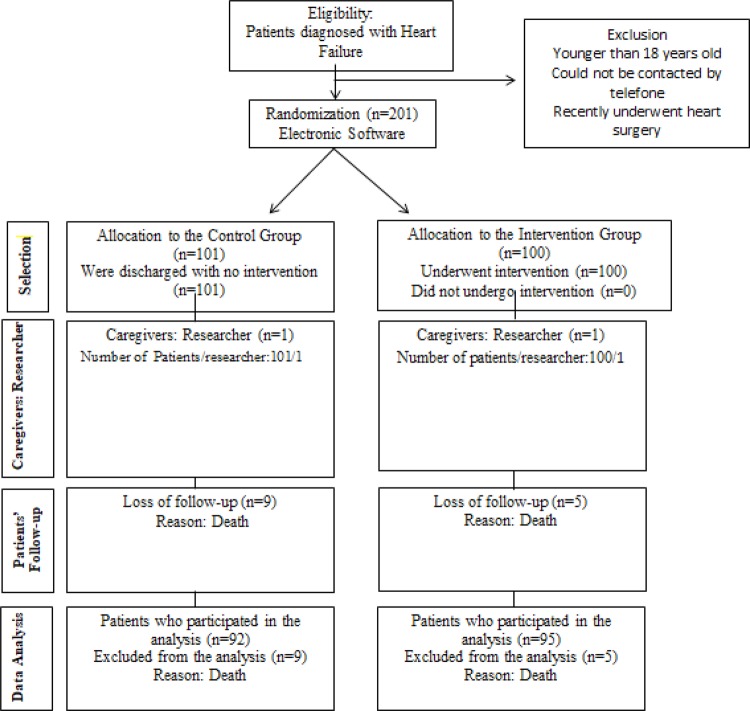
– Flowchart of the study. São Paulo, SP, Brazil, 2016

The flowchart of the study is shown in Figure 2.

## Results

The average age of the patients (n=201) was 62.6±15.2 years old, and the intervention and control groups were homogeneous with regard to the socio-demographic and clinical variables (p > 0.05), as noted in Table 1.

**Table 1:** – Demographic and clinical variables of the patients in the Intervention and Control Groups. São Paulo, SP, Brazil, 2016

Characteristics	Intervention Group	Control Group	P value*
	n(%)	n(%)	
*Sex*			0.527
Female	57 (57)	62 (61.4)	
Male	43 (43)	39 (38.6)	
*Nonsmoker*	42 (42)	33 (32.6)	0.076
*Former smoker*	29 (29)	38 (37.7)	0.076
*Smoker*	29 (29)	30 (29.7)	0.076
*Diabetes Mellitus*	59 (59)	60 (59.4)	0.357
*Dyslipidemias*	76 (76)	61 (60.4)	0.142
*SAH* ^†^	97 (97)	101 (100)	0.316
*Education level*			0.768
Five to eight years of study	36 (36)	39 (38.6)	
Illiterate	5 (5)	7 (6.9)	
*Occupation*			0.620
Retirees	28 (28)	20 (19.8)	
Homemaker	23 (23)	27 (26.7%)	

*P value: Chi-Square Test, Likelihood-Ratio Test, Mann-Whitney Test; †SAH: Systemic Arterial Hypertension

The medications were defrayed integrally by 45.8% (n = 92) of the patients, and 36.8% (n = 74) received all medications freely.

Adherence and the barriers to drug and non-drug adherence are presented in Table 2.

**Table 2:** – Drug adherence, barriers to drug and non-drug adherence between the intervention and control groups after 90 days of the intervention. São Paulo, SP, Brazil, 2016

Adherence/Barriers	Intervention Group (n=98)/ n(%)	Control Group (n=95)/ n(%)	Total n (%)	P value*	RR^†^	95% CI^‡^
**Drug Adherence**						
High adherence	24 (25.3)	7 (7.6)	31 (16.6)	**0.0003**	3.80	[1.77: 8.12]
Moderate adherence	32 (33.7)	55 (59.8)	87 (46.5)			
Low adherence	39 (41.1)	30 (32.6)	69 (36.9)	0.6124	1.56	[0.09: 2.22]
**Regimen Barrier**						
Present	56 (58.9)	72 (78.3)	128 (68.4)	**0.0045**	1.89	[1.2:2.98]
Absent	39 (41.1)	20 (21.7)	59 (31.6)			
**Belief Barrier**						
Present	15 (15.8)	32 (34.8)	47 (25.1)	**0.0028**	1.29	[1.09:1.53]
Absent	80 (84.2)	60 (65.2)	140 (74.9)			
**Memory Barrier**						
Present	37 (38.9)	61 (66.3)	98 (52.4)	**0.0002**	1.81	[1.3:2.52]
Absent	58 (61.1)	31 (33.7)	89 (47.6)			
**Non-Drug Adherence**						
High adherence	24 (25.3)	7 (7.6)	31 (16.6)	**0.0335**	1.94	[1.15:3.28]
Moderate adherence	32 (33.7)	55 (59.8)	87 (46.5)			
Low adherence	39 (41.1)	30 (32.6)	69 (36.9)		1.23	[0.78:1.94]

*P value: Generalized Estimating Equations Model; †RR: Relative Risk; ‡CI: Confidence Interval

In relation to drug adherence, there was statistically significant difference between the CG and the IG 90 days after hospital discharge. IG patients showed higher drug adherence, being 3.8 more likely to adhere when compared to patients of the CG in the same period.

As for the barriers to adherence in both CG and IG, the regimen barrier was the most prevalent, with statistically significant difference between the CG and the IG in relation to the presence of belief, regimen and memory barriers, which were more present in the CG, the IG being 1.89 times more likely to have negative barriers when compared to the CG.

With regard to non-drug adherence, there is a significant difference between the groups in relation to Adherence and Likely Adherence. The IG has a higher percentage of Adherence compared to the CG, being 1.2 times more likely of being Adherent than the CG after the intervention.

Regarding re-hospitalization, there was statistically significant difference (p<0.0001) between the two groups 90 days after the intervention. The IG has a lower re-hospitalization rate when compared to the CG. As for mortality, the IG has a lower death rate when compared to the CG, as noted in Table 3.

**Table 3:** – Re-hospitalization and deaths in the study population after seven, 30 and 90 days of the intervention. São Paulo, SP, Brazil, 2016 (n=201)

Outcomes	Control Group n (%)	Intervention Group n (%)	Total n (%)	P value*	RR^†^	95% CI^‡^
**30 days**						
**Re-hospitalization**						
No	96 (95)	98 (98)	194 (96.5)	0.4448	2.48	[0.49:12.46]
Yes	5 (5)	2 (2)	7 (3.5)			
**Death**	–	–	–			
**90 days**						
**Re-hospitalization**						
No	53 (52.5)	69 (69)	122 (60.7)	**0.0310**	1.55	[1.03:2.32]
Yes	39 (38.6)	26 (26)	65 (32.3)			
**Death**						
No	92 (91.1)	95 (95)	187 (93)	0.2761	1.78	[0.62: 5.13]
Yes	9 (8.9)	5 (5)	14 (14)			

*P value: Generalized Estimating Equations Model; †RR: Relative Risk; ‡CI: Confidence Interval

During the study period, the losses were due to the patients’ mortality, there having been no other reasons for withdrawal.

## Discussion

The results of this study showed that there is statistically significant difference with regard to drug and non-drug adherence between patients undergoing specific discharge guidance and telephone follow-up (IG) and patients undergoing standardized hospital guidance (CG). The IG patients showed more adherence to drug and non-drug treatment when compared to the other group. These results are consistent with the literature, since the nurse, by implementing educational practices in health such as nursing consultations, home visits and telephone follow-up, can establish appropriate interventions for drug and non-drug treatment, improving the understanding of the disease, adherence to treatment, self-monitoring of decompensation signs and symptoms, and self-care in relation to HF^15^.

The main purpose of nursing education for HF patients is that they understand the importance of adherence to treatment and the signs and symptoms of decompensation. Education in health, promoted by different strategies, can positively impact the treatment of HF; both telephone follow-up and domiciliary visits have been proven as effective to increase the adherence and self-care of this population^16^.

With respect to barriers to drug adherence, this study showed that the main one was the regimen barrier, suggesting the patients’ difficulty in understanding the guidelines on the prescription of medicines, which is worsened by polypharmacy^6^.

In relation to the belief barrier, which evaluates to what extent the patient believes the treatment may improve or control the disease, it was evidenced that the CG had a higher belief barrier percentage in relation to the IG. It can be inferred that after the intervention, the patients had better knowledge of the disease and of the importance of treatment, contributing to their belief in its effectiveness, which can improve their self-management of the disease and increase their adherence to the proposed therapy^7^. As for the presence of the memory barrier, a higher percentage was observed in the CG, which suggests that, after undergoing an educational intervention on the importance of treatment, individuals are less likely to forget about the medications and how to use them.

In this study, 16.6% of the total patients had high non-drug adherence, with difference between the groups: 7.6% of the CG and 25.3% of the IG. Non-drug adherence consists in suitable water restriction when indicated, restriction of salt intake and daily control of weight, and can be referenced as the target that is most difficult to be achieved in the treatment of HF, as it is related to changes of behavior and life habits^17^. A systematic review that examined 17 studies on self-care and chronic diseases, including HF, corroborates the idea that the patient’s self-care in relation to the disease is directly related with the decrease in mortality and re-hospitalization rates, needing thus to be addressed in education in health^18^.

The adherence to treatment of chronic patients has been shown to be a critical issue in the field of health^19^, as low adherence is directly related to re-hospitalization due to HF. The interventions carried out in this study contributed to lower re-hospitalization rates 90 days after the patients had been discharged, as 38.6% of the patients in the CG and 26% of those in the IG were hospitalized during this period (p=0.0310).

In relation to mortality, in this study, 7% of the total patients died, 8.9% in the CG and 5% in the IG, 90 days after discharge. A study assessing the impact of telephone follow-up on patients with HF also showed reduction in the mortality rate of these individuals^19^. Education in health, when carried out appropriately, can directly and significantly influence the mortality of patients with HF by improving these patients’ understanding of the disease, its treatment and, consequently, their adherence to therapy, with increased survival rates and improvements in their quality of life^19-20^.

This study had as limitation the patients’ follow-up period. Although most of the existing studies carry out follow-up for 90 days, a longer period could elucidate different outcomes and enable better evaluation regarding the treatment of HF. The dual educational strategy, which consisted of specific discharge guidance and telephone follow-up, proved to be beneficial in relation to the outcomes assessed in this study, but their association does not allow evaluating the effectiveness of each individual strategy, creating the need for studies on these strategies in isolation, and with increased follow-up time.

This study demonstrated the important role of the guidance provided by nurse, its impact on the adherence to the HF treatment and on the decrease in the number of re-hospitalization and death rates. In addition, randomized and controlled clinical trials may serve as a guide and reference for decision making in nursing practice. These findings may contribute substantially to the implementation of an individualized routine of discharge guidance and follow-up of patients with HF in other institutions as well, decreasing the demand for emergency services, the number of hospitalizations and deaths, and the cost to the healthcare system.

## Conclusion

This research concluded that the dual educational intervention, with guidance at the moment of and telephone follow-up after discharge of patients with HF in an emergency service is effective, and resulted in greater therapeutic adherence and reduction of barriers to it, in addition to decreasing re-hospitalization and mortality rates, as proposed.
